# TGF-β1 increases viral burden and promotes HIV-1 latency in primary differentiated human bronchial epithelial cells

**DOI:** 10.1038/s41598-019-49056-6

**Published:** 2019-08-29

**Authors:** S. Chinnapaiyan, R. K. Dutta, M. Nair, H. S. Chand, I. Rahman, H. J. Unwalla

**Affiliations:** 1 Department of Immunology and Nano-Medicine, Herbert Wertheim College of Medicine, Florida International University, Miami, FL 33199 USA; 2 University of Rochester Medical Center, School of Medicine and Dentistry, Rochester, NY 14642 USA

**Keywords:** Infection, Immunological deficiency syndromes

## Abstract

Combination antiretroviral therapy (cART) has increased the life expectancy of HIV patients. However, the incidence of non-AIDS associated lung comorbidities, such as COPD and asthma, and that of opportunistic lung infections have become more common among this population. HIV proteins secreted by the anatomical HIV reservoirs can have both autocrine and paracrine effects contributing to the HIV-associated comorbidities. HIV has been recovered from cell-free bronchoalveolar lavage fluid, alveolar macrophages, and intrapulmonary lymphocytes. We have recently shown that *ex*-*vivo* cultured primary bronchial epithelial cells and the bronchial brushings from human subjects express canonical HIV receptors CD4, CCR5 and CXCR4 and can be infected with HIV. Together these studies suggest that the lung tissue can serve as an important reservoir for HIV. In this report, we show that TGF-β1 promotes HIV latency by upregulating a transcriptional repressor BLIMP-1. Furthermore, we identify miR-9-5p as an important intermediate in TGF-β-mediated BLIMP-1 upregulation and consequent HIV latency. The transcriptionally suppressed HIV can be reactivated by common latency reactivating agents. Together our data suggest that in patients with chronic airway diseases, TGF-β can elevate the HIV viral reservoir load that could further exacerbate the HIV associated lung comorbidities.

## Introduction

In aging HIV-infected populations, comorbid diseases are important determinants of morbidity and mortality. Even in the cART era, HIV patients die of non-AIDS comorbidities almost a decade earlier than their non-HIV counterparts^1^. cART successfully suppresses viral replication but is unable to eradicate HIV due to presence of latently infected anatomical reservoirs^2,3^. As people living with HIV grow older, the long-term effects of low-level viral replication and expression of viral proteins from these reservoirs, as well as treatment with cART itself, promotes many of these comorbidities. For instance, HIV Tat, secreted by infected cells, can have pleiotropic effects on neighboring cells as well. HIV Tat is an immediate early gene of HIV and its expression is not suppressed by antiretrovirals^1–4^. Lung diseases such as COPD, are emerging as significant comorbidities in the HIV-infected population^5,6^. A large proportion of people living with HIV also smoke tobacco that can exacerbate lung comorbidities^7^. HIV infection is an independent risk factor for the development of COPD even after accounting for smoking status^8^. Increased HIV reservoir load in the lung can exacerbate HIV associated lung comorbidities. We have shown that primary human bronchial epithelial cells can be infected with HIV^9^. The infection kinetics demonstrated an early spike in virus release that subsides and reaches a low-level steady state with detectable HIV Gag and HIV RNA up to 50 days post-infection (duration of experiment) suggesting that bronchial epithelium can serve as HIV reservoirs.

TGF-β is a multifunctional growth factor and it regulates cell growth, adhesion and differentiation in a wide variety of cell types^10,11^. TGF-β isoforms are expressed and secreted by several cell types in the airway, including epithelia in association with a latency-associated peptide. This provides a TGF-β reservoir in the extracellular matrix^12^ that participates in normal lung physiological processes and functions in local immunomodulation, regulation of cell proliferation and differentiation, as well as the control of normal tissue repair. TGF-β signaling is upregulated by HIV Tat, cigarette smoke and in chronic lung diseases like COPD, asthma, pulmonary fibrosis and lung infections^13–19^. We have previously reported that TGF-β and cigarette smoke suppress miR-141-5p to promote CCR5 expression on primary bronchial epithelial cells, which increases viral entry and infection by R5-tropic HIV. In this report, we demonstrate that TGF-β signaling downregulates miR-9-5p, thereby increasing expression of the transcriptional repressor BLIMP-1, known to promote formation of HIV reservoirs^20^. We show that TGF-β induced BLIMP-1 expression correlates with HIV transcriptional suppression in primary bronchial epithelial cells. Taken together with our earlier reports, TGF-β signaling in the airway promotes infection of bronchial epithelium and increases the reservoir pool in the airway.

## Results

### TGF-β promotes HIV latency in NHBE cells

**Figure 1:**
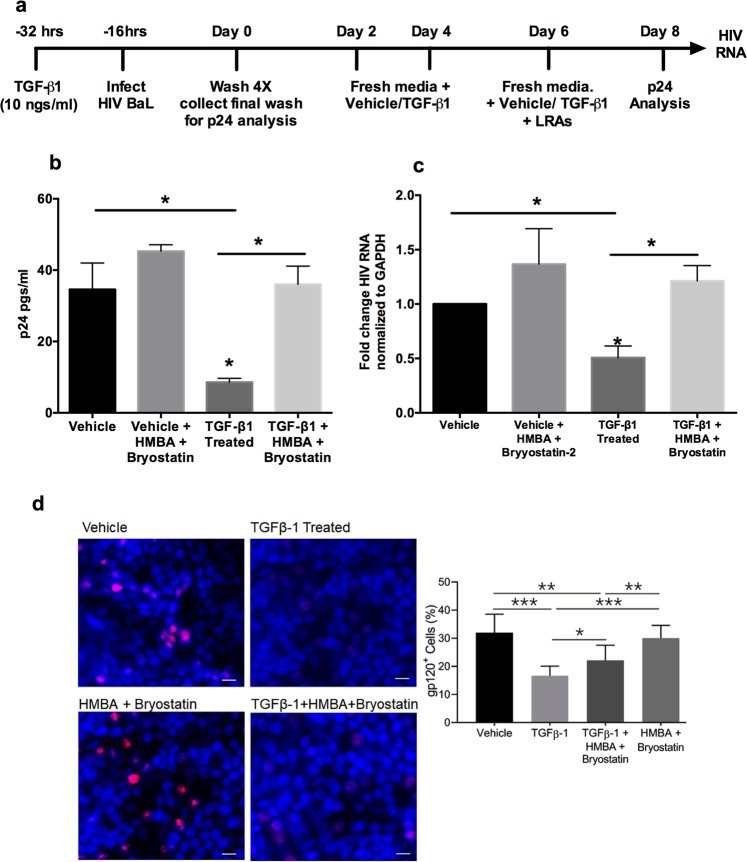
TGF-β1 promotes HIV latency in NHBE cells. (**a**) Experimental timeline for effect of TGF-β1 on HIV infection. (**b**) NHBE cultures redifferentiated at the ALI were treated and infected as shown in (**a**). Culture supernatants were collected on day 8 for post-reactivation p24 analysis using p24 ELISA kit. (**c**) Total RNA was isolated and HIV transcripts were quantitated by qRT-PCR. TGF-β1 treatment suppresses HIV mRNA and this can be reversed with the combination of LRAs Bryostatin-2 and HMBA. TGF-β1 suppresses viral p24 and this corelates well with mRNA suppression. n = NHBE ALI cultures from at least 5 different lungs; *p < 0.05. (**d**) The TGF-β-mediated suppression of gp120 expression levels in HIV-infected and latency reactivated NHBEs, scale – 10 µ. n = NHBE ALI cultures from 3 different lungs, *p < 0.05).

TGF-β signaling alters the bronchial epithelial microRNAome^21^. Several miRNAs are known to directly or indirectly affect HIV replication^22^. In our earlier report, we have identified that cigarette smoke and TGF-β1 increases the expression of CCR5 coreceptor by suppressing miR-141-5p that, at least in case of cigarette smoke, increases entry by R5-tropic HIV BaL strain^23^. Given that TGF-β signaling is upregulated by Tat, cigarette smoke and in chronic lung diseases, we tried to determine the effects of persistent TGF-β signaling on HIV infection in primary bronchial epithelium redifferentiated *ex*-*vivo*. NHBE cultures redifferentiated at the ALI were pretreated with recombinant TGF-β1 (10 ng/ml; vehicle as control). Following sixteen hours of pretreatment (based on our data that effects of TGF-β1 effects changes in miR-141-5p and CCR5 expression after 16 hours of treatment^21^), NHBE ALI cultures were infected with 5 ng p24 equivalent of HIV BaL (R5-tropic strain) as described by us earlier^9,23^. An additional 16 hours post-infection, cultures were washed apically and basolaterally four times with phosphate buffer saline (PBS). The fourth PBS wash was collected as day 0 to confirm that all input virus had been removed. Media was completely replaced every 48 hours with fresh ALI media with vehicle or TGF-β. A schematic of the treatment regimen is demonstrated in Fig. 1a. Poli *et al*. have demonstrated that acute TGF-β1 treatment interferes with early transcription of HIV in U1 cell lines^24^. To determine if TGF-β1 promotes latency, an identical experimental set was treated with latency reactivating agents (LRAs), Bryostatin-2 (10 nM/ ml; Protein kinase C agonist), and HMBA (10 mM/ ml; P-TEFb activator) on Day 6. Darcis *et al*.^25^, have shown that a combination of prostatin/HMBA followed by Bryostatin-2/HMBA provides maximal transcriptional reactivation across multiple cells lines tested. However, prostatin also downregulates the CCR5 receptor^26^, which can directly contradict the effects of TGF-β1 mediated CCR5 upregulation on de-novo HIV infection. Hence, we chose a combination of Bryostatin-2/HMBA as LRAs. The infections were allowed to proceed up to day 8. Culture supernatants were collected and analyzed for HIV p24. As seen in Fig. 1b, TGF-β1 treatment decreases supernatant p24. Addition of LRAs, Bryostatin-2/HMBA to TGF-β1 treated NHBE cells restores p24 levels. To confirm if this suppression is a consequence of a direct effect on viral transcription and not due to indirect effects of the LRAs on cellular co-factors involved in later stages of viral replication, total RNA from an identical experiment was isolated and HIV transcripts were quantitated by qRT-PCR as described by us earlier^9^. As seen in Fig. 1c, TGF-β1 suppresses viral RNA and this suppression correlates with HIV p24 suppression. Addition of HMBA/Bryostatin-2 to HIV BaL infected NHBE cells, not treated with TGF-β1, did not demonstrate significant increase in viral RNA or p24 levels. Together these data demonstrate that that TGF-β suppresses viral transcription and promotes HIV latency in NHBE ALI cultures. The experimental regimen had only a marginal effect on cell viability across all treatments on Day 8 (Supplementary Fig. 2).

Next, we determined whether TGF-β1 increases the number of cells infected by HIV BaL (R5-tropic strain). NHBE ALI cultures were treated and reactivated identically. Cultures were fixed and stained with anti-gp120 antibody as described before^27^. As seen in Fig. 1d, TGF-β1 suppresses intracellular expression of HIV gp120. However, addition of LRAs demonstrates an increase in intensity as well as number of cells stained with anti-gp120 antibody. Together, these data suggest that TGF-β1 enhances infection of NHBE ALI cultures but suppresses HIV transcription.

### TGF-β signaling alters mRNA levels of HIV-1 host restriction factors in NHBE cells

**Figure 2:**
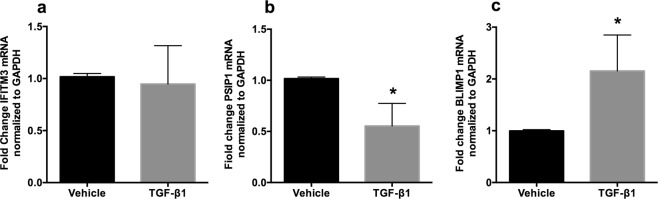
TGF-β signaling alters mRNA expression of HIV-1 host restriction factors in NHBE cells. NHBE ALI cultures were treated with recombinant TGF-β1 (10 ng/ml; vehicle as control). 48 hours post-treatment, total RNA was isolated and mRNA levels of IFITM3, PSIP1 and BLIMP-1 were determined by qRT-PCR. (**a**) TGF-β1 does not alter expression of IFITM3 mRNA. (**b**) TGF-β1 suppresses PSIP1 mRNA. (**c**) TGF-β1 increases mRNA levels of BLIMP-1 compared to vehicle treated controls. n = NHBE ALI cultures from 3 different lungs; *significant (p < 0.05).

Several reports have identified host cellular proteins that serve as anti-HIV restriction factors to block viral entry, nuclear import and integration, transcription, translation or budding. We had observed that TGF-β1 suppresses viral RNA and this relates well with HIV p24 output in Fig. 1. Given reports by Poli *et al*., that TGF-β1 suppresses early stage of HIV replication^24^, we tried to determine if TGF-β1 alters expression of cellular HIV restriction factors affecting viral entry, proviral DNA integration and transcription. We specifically focused on the expression of IFITM3 (virus-endosomal fusion), PSIP1/LEDGF/p75 (HIV integration) and BLIMP-1 (HIV proviral transcription). NHBE ALI cultures were treated with recombinant TGF-β1. Total RNA was isolated and mRNA levels of IFITM3, PSIP1 and BLIMP-1 were determined by qRT-PCR. As seen in Fig. 2a, TGF-β1 does not affect expression of IFITM3 but suppresses PSIP1 mRNA (Fig. 2b) and increases mRNA expression of BLIMP-1 (Fig. 2c), compared to vehicle treated controls.

### Changes in PSIP1 and BLIMP-1 mRNA levels manifest as decreases in protein levels

**Figure 3:**
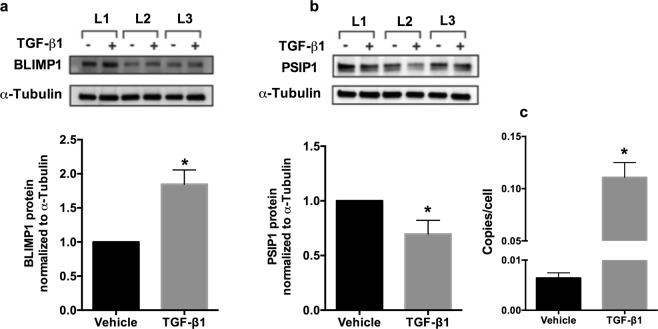
TGF-β1 alters the protein expression of HIV-1 restriction factors such as BLIMP-1 and PSIP-1 in NHBE cells. NHBE ALI cultures were treated with recombinant TGF-β1 (or vehicle as control). 48 hours post-treatment, total protein was isolated and quantified by western blot analysis. (**a**) BLIMP-1 protein levels were significantly higher in TGF-β1 treated NHBE ALI cultures compared to vehicle treated controls. (**b**) TGF-β1 suppresses PSIP-1 protein levels in NHBE ALI cultures compared to vehicle treated controls. (**c)** TGF-β1 treatment demonstrates increased HIV integration events in NHBE cells. NHBE ALI cultures were treated with recombinant TGF-β1 (or vehicle as control). 16 hours post-treatment NHBE ALI cultures were infected with 5 ng p24 equivalent of HIV BaL (R5-tropic strain) and the infection was allowed to proceed for an additional 24 hours. Experiments were terminated and genomic DNA was isolated and used to quantitate integrated HIV proviral DNA. n = NHBE ALI cultures from 3 different lungs; *significant (p < 0.05). L = Lung number.

Next, we tried to determine if TGF-β induced changes in BLIMP-1 and PSIP1 mRNA translates to changes in their protein levels as well. NHBE ALI cultures were treated with TGF-β1. 48 hours post-treatment, total protein was isolated and quantified by western blot analysis for BLIMP-1 and PSIP1 expression. We found that TGF-β mediated changes in mRNA expression of BLIMP-1 and PSIP1 translates to corresponding changes in protein levels as well. TGF-β signaling significantly upregulates BLIMP-1 protein levels (Fig. 3a) and suppresses PSIP1 levels (Fig. 3b) compared to vehicle treated controls. Next we tried to determine if suppression of PSIP1 translates to decreased proviral integration. NHBE ALI cultures were treated with recombinant TGF-β1 (or vehicle as control). Sixteen hours post-treatment NHBE ALI cultures were infected with 5 ng p24 equivalent of HIV BaL, and the infection was allowed to proceed for an additional 24 hours. Experiments were terminated and genomic DNA was used to quantitate integrated HIV proviral DNA as first demonstrated by Brussel and Sonigo^28^ and later adapted by us^9^. As seen in Fig. 3c, TGF-β1 mediated suppression of PSIP1 does not result in decreased viral integration, suggesting that the magnitude of TGF-β mediated PSIP1 suppression is not sufficient to affect viral integration. On the contrary, TGF-β1 significantly increases the number of integrated provirus in NHBE ALI cultures.

### BLIMP-1 and Histone deacetyl transferases are involved in TGF-β induced latency in the bronchial epithelium

**Figure 4:**
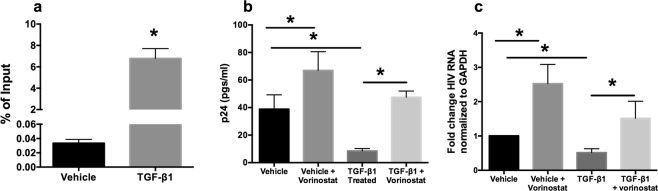
TGF-β1 mediates HIV suppression via BLIMP-1 and histone deacetylases. NHBE ALI cultures were treated with TGF-β1 or vehicle and infected similar to that in Fig. 1a. Chromatin was isolated on Day 3 and BLIMP-1 mobilization to HIV LTR was determined using CHIP. TGF-β1 increases mobilization of BLIMP-1 to HIV LTR (**a**). n = NHBE ALI cultures from 4 different lungs; *significant (p < 0.05). Another set was TGF-β1 treated and infected identically, On day 6 the HDAC inhibitor vorinostat (1 μM) was added. On day 8 culture supernatants were collected and analyzed for HIV p24 (**b**). Total RNA was isolated and analyzed for HIV LTR using qRT-PCR (**c**). TGF-β1 mediated HIV transcriptional suppression can be restored by HDACi suggesting a role of histone deacetylases in TGF-β1 mediated HIV latency. n = NHBE ALI cultures from 5 different lungs; *significant (p < 0.05).

BLIMP-1 is a transcriptional repressor that can potentially suppress HIV transcription via multiple pathways. Indeed BLIMP-1 has been shown to directly bind HIV LTR^20^. However, BLIMP-1 has also been shown to mediate transcriptional repression via HDAC recruitment^29^ and suppression of transcription factors like Myc, which can indirectly impact HIV transcription^30^. This is further complicated by reports that BLIMP-1 itself is regulated by HDACs^31^ and HDAC inhibitors suppress BLIMP-1^32^. BLIMP-1 has been shown to directly activate 93 different genes and directly suppresses 121 target genes. Of these, BLIMP-1 activated 7 and repressed 13 transcription factors, any of which may impact viral transcription^33^. To determine if the effects of TGF-β1 mediated suppression is a consequence of direct binding of BLIMP-1 to HIV LTR, we performed a chromatin immunoprecipitation assay (CHIP) as described^20^. NHBE ALI cultures were pretreated with TGF-β1 and infected with 5 ng p24 equivalent of HIV BaL as mentioned in Fig. 1. Eight days post-infection, chromatin was prepared and protein-DNA complexes were precipitated and enriched with Blimp-1-specific antibody. Michaels *et al*.^20^, have reported BLIMP-1 binding at +142/+237 ISRE site in HIV LTR. Figure 4a shows that TGF-β1 treatment significantly increases BLIMP-1 mobilization at this site in NHBE ALI cultures infected with HIV BaL. BLIMP-1 has been shown to mediate its effects via recruitment of histone deacetylases^29^. Moreover BLIMP-1 itself is epigenetically regulated^31,32^. NHBE ALI cultures were treated similar to that in Fig. 1a. On day 6, the HDAC inhibitor vorinostat (1 μM) was added to the cultures. Vorinostat restores HIV transcription in TGF-β1 treated cells and this is reflected in viral p24 levels identifying the role of HDACs in TGF-β1 induced HIV latency (Fig. 4b,c). Surprisingly we observed that vorinostat also increased HIV output in TGF-β1 untreated (vehicle treated) NHBE ALI cultures suggesting that there is baseline suppression of HIV transcription in primary bronchial epithelial cells by HDACs. Hence primary bronchial epithelium can serve as HIV reservoirs and this is exacerbated by TGF-β1 signaling.

### TGF-β1 upregulates BLIMP-1 by suppressing miR-9-5p

TGF-β signaling has been shown to alter miRNA homeostasis^34,35^. In our previous report, we have demonstrated that TGF-β suppresses miR-141-5p to increase expression of CCR5 receptor. We tried to determine if TGF-β mediated BLIMP-1 upregulation is a consequence of miRNA mediated regulation. Analysis of literature revealed a potential link between TGF-β signaling, miR-9-5p and BLIMP-1. miR-9-5p has been shown to regulate BLIMP-1^36,37^, while TGF-β has been shown to epigenetically suppress miR-9-5p expression^38^. In our recent report, a microRNAome analysis of TGF-β1 treated NHBE ALI cultures demonstrated a Log2 fold suppression of −4.45 in expression of miR-9-5p^39^. We used a systematic approach to identify the role of miR-9-5p in TGF-β mediated BLIMP-1 upregulation.

**Figure 5:**
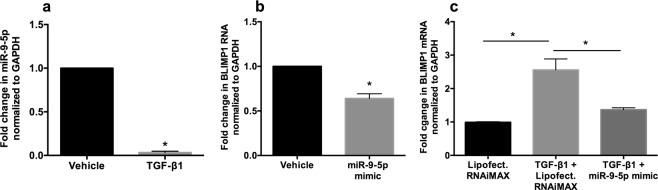
TGF-β1 upregulates BLIMP-1 by suppressing miR-9-5p. NHBE ALI cultures were treated with recombinant TGF-β1 (or vehicle as control). (**a**) TGF-β1 completely abolishes miR-9-5p expression in NHBE ALI cultures compared vehicle treated control. n = NHBE ALI cultures from three different lungs. (**b**) BEAS-2B airway epithelial cells were transfected with miR-9-5p mimic (20 nM) using lipofectamine RNAiMAX according to manufacturer’s instructions. 48 hours post-transfection, total RNA was isolated and quantitated for BLIMP-1 expression. The miR-9-5p mimic suppress BLIMP-1 mRNA compared to lipofectamine RNAiMAX alone treated cells. n = 3 different experiments using BEAS-2B cells. (**c**) BEAS-2B airway epithelial were transfected with miR-9-5p mimic (20 nM) using lipofectamine RNAiMAX (or lipofectamine RNAiMAX as control). 24 hours following transfection, cells were treated with recombinant TGF-β1. 16 hours following TGF-β1 treatment, total RNA was isolated and quantitated for BLIMP-1 expression. The TGF-β1 increases BLIMP-1 expression and this increase is reversed in cells transfected with miR-9-5p mimic. n = 3 different experiments using BEAS2B cells. *significant (p < 0.05).

We first tried to validate our miRNA array data to determine if TGF-β suppresses miR-9-5p in primary bronchial epithelial cells. NHBE ALI cultures were treated with recombinant TGF-β1 (vehicle as control). As seen in Fig. 5a, TGF-β1 completely abrogates miR-9-5p expression in NHBE ALI cultures. Next, we tried to determine if miR-9-5p suppresses BLIMP-1. For these experiments, we used transient transfection of the miR-9-5p mimic in BEAS-2B airway epithelial cells. This is because BEAS-2B cells are readily transfected with a higher transfection efficiency compared to primary bronchial epithelial cells. BEAS-2B airway epithelial cells were transfected with miR-9-5p mimic (20 nM) using lipofectamine RNAiMAX according to manufacturer’s instructions. Forty-eight hours post-transfection, total RNA was isolated and quantitated for BLIMP-1 expression. As seen in Fig. 5b, miR-9-5p mimic suppresses BLIMP-1 mRNA. Next, we tried to determine if miR-9-5p mimics can reverse TGF-β mediated BLIMP-1 upregulation. BEAS-2B airway epithelial were transfected with miR-9-5p mimic (20 nM) using lipofectamine RNAiMAX. 24 hours following transfection, cells were treated with recombinant TGF-β1. Lipofectamine RNAiMAX was added to the control set and was also included in the TGF-β1 treated set. Sixteen hours following TGF-β1 treatment, total RNA was isolated and quantitated for BLIMP-1 expression. As seen in Fig. 5c, TGF-β signaling increases BLIMP-1 expression and this increase is reversed in cells transfected with miR-9-5p mimic.

## Discussion

HIV infection is linked with a greater incidence of pulmonary diseases normally associated with aging and their presentation at younger ages. cART cannot eradicate HIV due to the presence of HIV reservoirs in different anatomical sites^40^. Persistent low-level viral replication coupled with expression of viral proteins not controlled by antiretrovirals are the principal causes of non-AIDS comorbidities in people living with HIV^41,42^. The onset and severity of these comorbidities can correlate with the viral reservoir load^43^. Airway diseases like chronic bronchitis, pneumonia, asthma and COPD are principal non-AIDS HIV associated comorbidities in people living with HIV. In our previous studies, we first demonstrated that the bronchial epithelium expresses canonical HIV receptors CD4, CCR5 and CXCR4 and can be infected by both R5 and X4 tropic HIV. Several reports have shown that TGF-β can have contradictory effects on HIV transcription in different cell types^44,45^ while also having contradictory effects on different pathways of HIV activation. For instance, TGF-β suppresses PMA induced activation but not TNF-α induced activation of HIV LTR^24^. Given that, TGF-β signaling is increased in smokers and chronic airway diseases, we tried to determine the effects of increased TGF-β signaling on HIV infection. In our previous report we have shown that HIV output spikes initially followed by a gradual decrease that reaches steady state around Day 8^9^. To disentangle the effects of HIV suppression by TGF-β1 from the normal kinetics of decreasing viral output observed in NHBE cells, we chose addition of LRAs on day 6 followed by culture supernatant p24 readout on day 8. Our data demonstrate that TGF-β treatment suppresses HIV RNA, which manifests as decreased viral output and this can be reversed by some of the common latency reactivating agents. While our earlier manuscript reported some cell death around Day 6 which is exacerbated by Day 12, this was observed using the RGH-WT virus which is env negative and expresses GFP. The virus was packaged in *in vitro* transient transfections of HEK293 cells with the virus plasmid DNA and either the R5 or X4 tropic HIV envelope. Given that this was a single cycle infection we had also used a higher dose for infection (10 ngs p24 equivalent compared to 5 ng used in the current study). Our experimental design did not significantly affect cell viability on day 8 across all treatments. Transcriptional suppression is considered one of the principal mechanisms of HIV latency^46,47^.

Next, we analyzed the effect of TGF-β signaling on some of the common cellular restriction factors that affect viral entry, integration and transcription. Our data showed that TGF-β alters the expression of two of the restriction factors tested. TGF-β1 decreases PSIP1 mRNA and protein levels, involved in viral integration, and increases the expression of BLIMP-1, a transcriptional repressor known to facilitate HIV latency. To determine if decreased HIV RNA and p24 levels were a consequence of decreased integration or suppressed transcription or both, we quantitated the integrated proviral DNA in cells treated with TGF-β. Our data (Fig. 3c) showed that TGF-β treated NHBE cultures demonstrated increased integration events suggesting that the magnitude of PSIP1 suppression is not sufficient to observe discernible effects on viral integration. Moreover, increased proviral DNA could also be a consequence of increased viral entry on account of CCR5 upregulation. Our data agree with prior observations that even a 90% suppression of PSIP1 using RNA interference had only modest effects on viral infectivity^48,49^ while only a complete knockdown blocks viral integration^50^. Interestingly, the magnitude of increase in integrated copies correlates very well to increase in viral entry by cigarette smoke mediated upregulation of CCR5 and consequently HIV entry in cigarette smoke exposed NHBE ALI cultures^28^. BLIMP-1 expression is increased in chronically infected HIV patients and correlates with enhanced expression of negative regulators of T cell activation including PD-1, LAG3 and CTLA-4, and with T cell exhaustion and apoptosis^36,51^. Furthermore, the HIV-1 long terminal repeat (LTR) includes binding sites for BLIMP-1^52^. While it is possible that being a master regulator of transcription, BLIMP-1 could also mediate its effects indirectly as a consequence of altered expression of cellular cofactors. Our data demonstrates that TGF-β1 mediates HIV latency at least in part due to a direct mobilization of BLIMP-1 to the HIV LTR. Moreover, Vorinostat can restore TGF-β1 mediated HIV latency suggesting that BLIMP-1 may be mediating its effects via histone deacetylases.

We have shown that TGF-β1 alters the bronchial epithelial microRNAome^21^ Other reports have also shown that TGF-β signaling alters miRNA homeostasis in different cell types^34,35^. We recently reported that TGF-β signaling suppresses the expression of miR-9-5p^21^. A number of reports in literature identified miR-9-5p as one of the miRNAs regulating BLIMP-1^36,37^. Likewise, miR-9-5p and TGFβ are also involved in an autoregulatory feedback loop where miR-9-5p inhibits TGF-β signaling and vice versa^38^. As a pilot experiment, we revisited our RNA samples from TGF-β treated cells to determine levels of miR-9-5p. As seen in Fig. 5a, TGF-β treated NHBE ALI cultures demonstrated a complete abrogation of miR-9-5p expression. Next, we confirmed that miR-9-5p suppresses BLIMP-1 expression and miR-9-5p mimics can reverse the effects of TGF-β1 on BLIMP-1 mRNA (Fig. 5b,c), thereby confirming that TGF-β1 suppresses miR-9-5p to upregulate its target BLIMP-1. This also provides proof-of-concept that miR-9-5p antagomiRs can be used to modulate TGF-β signaling in the airways and reverse TGF-β induced HIV latency in the airway. While our data has explored HIV latency in bronchial epithelial cells, it is possible that TGF-β increases viral reservoirs in other cell types in the lung like alveolar macrophages by a similar mechanism.

**Figure 6:**
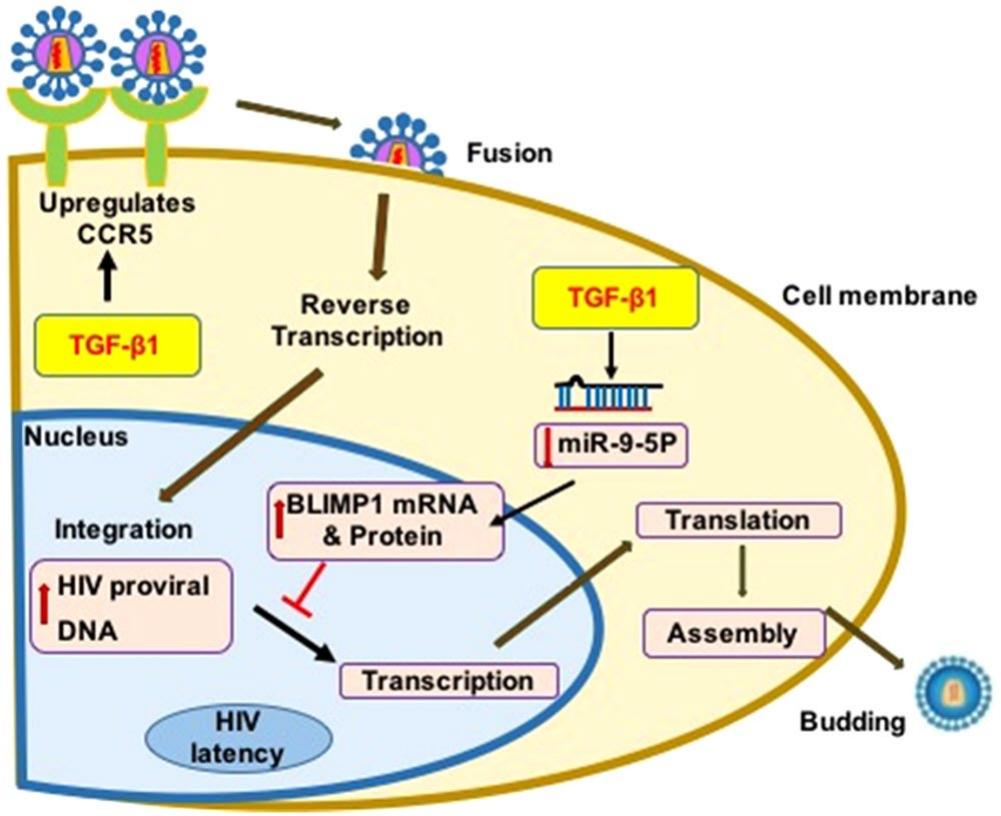
TGF-β1 increases HIV reservoir load in the bronchial epithelium. Schematic representation of the effects of TGF-β1 on host restriction factors to promote the HIV-1 reservoir load in human bronchial epithelial cells. TGF-β1 increases levels of canonical HIV receptor CCR5. This increases infection of bronchial epithelium by R5-tropic HIV leading to an increased number of integrated HIV DNA. TGF-β1 also suppresses miR-9-5p with a consequent upregulation of its target transcriptional repressor BLIMP-1. Together this leads to increased infection events and latency thereby increasing the viral load in the airway.

In conclusion, we report for the first time that TGF-β1 altered bronchial epithelial microRNAome has a paradoxical effect on HIV infection of the bronchial epithelial cells resulting in an increased infection, while also promoting HIV latency. The mechanism is summarized in Fig. 6. TGF-β1 induced miR-141-5p increases CCR5 expression leading to an increased infection of airway epithelial cells while TGF-β1 induced miR-9-5p suppression upregulates BLIMP-1, which in turn suppresses viral transcription. The net effect of these actions will increase viral load while also promoting HIV latency. Inflammation during disease exacerbations will reactivate HIV transcription, consequently increasing the levels of HIV proteins in the airway. We and others, have already demonstrated that HIV proteins Tat and gp120 can affect components of the mucociliary clearance apparatus as well as epithelial barrier integrity^9^. Hence, increased viral load and in the airway will further exacerbate lung comorbidities in people living with HIV. Moreover, miR-9-5p might represent a promising and potentially novel biomarker as an index of TGF-β signaling and HIV viral load in the lungs and overall airway health in people living with HIV.

## Materials and Methods

### Cell culture

Primary normal human bronchial epithelial (NHBE) cells were obtained from properly consented donors whose lungs were not suitable for transplantation for the causes unrelated to airway complications and supplied by University of Miami Life Alliance Organ Recovery Agency (LAORA). Since the material was obtained from deceased individuals with minor, de-identified information, its use does not constitute human subjects research as defined by CFR 46.102. A signed and well-documented consent of everyone (or legal healthcare proxy) for donation of lungs for research purpose is on file with the LAORA and allows research purpose of this material. NHBE cells were isolated and re-differentiated at the air-liquid interface (ALI) cultures as described by Fulcher and Randall^53,54^ and adapted by us^55,56^. These primary cultures undergo differentiation at the ALI reproducing both the *in vivo* morphology and key physiologic processes to regenerate the native bronchial epithelium *ex vivo*^53,54^. Transformed human bronchial epithelial cell line BEAS-2B cells (CRL-9609, American Type Culture Collection ATCC, Manassas, VA, USA) were grown as monolayers at 5% CO_2_ at 37 °C in serum-free defined BEGM growth medium according to the supplier’s instructions.

### Virus strains and infection studies

The R5-tropic viral strain HIV BaL was a kind gift from Dr. Madhavan Nair (Department of Immunology and Nano-Medicine, Institute of Neuroimmune Pharmacology, Herbert Wertheim College of Medicine, Florida International University. Miami, FL). NHBE ALI cultures were infected apically and basolaterally with 5 ng p24 equivalent of R5-tropic HIV strain (BaL) as described earlier^9^. 16 hours post-infection, cells were washed apically and basolaterally with PBS four times to remove any residual input virus. The fourth wash was collected for p24 analysis and measured as day 0 to confirm that all input virus had been removed. Culture media was changed every 48 hours and media collected on Day 8 was used for p24 antigen analysis using p24 ELISA kit (ZeptoMetrix Corp. Cat # 0801200) according to manufacturer’s protocol.

### RNA isolation and quantitative real-time PCR (qRT-PCR)

To examine mRNA expression, total RNA was extracted using the Qiagen RNeasy mini kit (Cat # 74104) and complementary DNA (cDNA) was reverse transcribed using the Applied Biosystems high-capacity cDNA reverse transcription kit (Cat # 4368814). qRT-PCR was performed on the Bio-Rad CFX96 real-time system using validated TaqMan probes (Life Technologies/Applied Biosystems HIV-LTR, Cat # Pa03453409_s1; IFITM3, Cat # Hs03057129_s1; BST2, Cat # Hs01561315_m1; BLIMP-1, Cat # Hs00153357_m1; PSIP1, Cat # Hs01045711_g1; and GAPDH, Cat # Hs02786624_g1). qRT-PCR results are represented as relative quantification normalized against internal control (GAPDH).

### Detection of HIV proviral DNA

TGF-β1 (10 ng/ ml) treated with NHBE ALI cultures (vehicle as control) reported by us earlier^56^, it was purchased from the Life Technologies (Cat # PHG9214) and 16 hours post-treated cells were infected with 5 ng of HIV BaL (R5-tropic strain) HIV as described by us earlier^9^ and cells were washed with phosphate buffer saline (PBS) four times after 16 hours post-infection. After 24 hours, genomic DNA was extracted using the Wizard SV genomic DNA purification system (Promega, Cat# A2360) according to the manufacturer’s protocol. U937 cells (aidsreagent catalog # 165), stably infected with R5-tropic HIV, was used as integrated HIV DNA standard to generate the standard curve. HIV integration in NHBE ALI cultures was determined using comparative Ct method by 2-step qPCR method described by Brussel and Sonigo^28^ and adapted by us^9^. Cell equivalent was determined using the DNA copy number and dilution calculator (Thermofisher Scientific) based on amount of input DNA from infected NHBE ALI cultures and data was expressed as copies/cell.

### Western blot analysis and antibodies

Whole-cell lysates were prepared by washing cells with PBS and lysing them with radioimmunoprecipitation assay (RIPA) buffer (ThermoFisher Scientific, Cat # 89901) along with protease inhibitor cocktail (ThermoFisher Scientific, Cat # 78410). Protein was measured using the Bio-Rad protein assay dye reagent (Bio-Rad, Cat # 5000006) according to manufacturer directions. Briefly, each protein sample (50 µg) was subjected to heat denaturation at 100 °C for 5 minutes before loading onto a 4 to 20% gel and run at 100 V. Protein was transferred to PVDF membrane. Following blocking in 5% milk primary antibodies; BLIMP-1 (1:1000; Cell Signaling, Cat # 9115), PSIP1 (LEDGF/p75; 1:1000 Cell Signaling, Cat # 2088) or α-tubulin (1:1000; Cell Signaling, Cat # 2125) were added. Blot was incubated in an anti-rabbit secondary antibody diluted to a concentration of 1:2500. Bands were detected in Chemidoc (Bio-Rad Laboratories, USA) using supersignal west femto maximum sensitivity substrate (ThermoFisher Scientific, Cat # 34095) in accordance with the manufacturer’s instruction. Quantitative densitometry analyses were performed using the Quantity One software (Bio-Rad Laboratories, USA) and the density values are normalized to α-tubulin.

### Chromatin immunoprecipitations (ChIP)- qPCR

NHBE ALI cultures were treated with TGF-β1 (10 ng/ml; vehicle as control). 16 hours of post-treatment cultures were infected with HIV BaL as described by us earlier^9,23^. An additional 16 hours post-infection, cultures were washed apically and basolaterally four times with phosphate buffer saline (PBS) and this was considered Day 0. Day 3 post-infection cells were harvested and ChIP assay was performed using the ChromaFlash High-Sensitivity ChIP kit (Epigentek, Cat # P-2027) according to the manufacturer’s instructions. Chromatin samples were immunoprecipitated using anti-BLIMP-1 antibody (ChIP Grade, Abcam, Cat # ab13700). qPCR analyses were performed using EpiQuik Quantitative PCR Fast kit (Epigentek, Cat # P-1029) with primers 5′- TCCCTCAGACCCTTTTAGTCAG-3′ and 5′-GTCGAGAGAGCTCCTCTGGTTT-3′ flanking the +142 F/+237 R region of HIV-1 LTR reported by Michaels *et al*.,^20^. Data were analyzed as described by Natarajan *et al*.,^57^.

### miR-9-5p mRNA expression analysis by qRT-PCR

NHBE ALI cultures were treated with TGF-β1 (vehicle as control) as described above. Following 16 hours total RNA was isolated from control and TGF-β treated with NHBE ALI cultures by using the Qiagen RNeasy mini kit (Cat # 74104) and cDNA was reverse transcribed by the Applied Biosystems TaqMan™ Advanced miRNA cDNA synthesis kit (Life Technologies/Applied Biosystems, Cat # A28007) according to the manufacturer’s instruction. qRT-PCR was done using TaqMan™ fast advanced master mix (Life Technologies/Applied Biosystems, Cat # 4444557) in combination with validated TaqMan probes (Life Technologies/Applied Biosystems, hsa-miR-9-5p, Cat # A25576, 478214_mir) according to the manufacturer’s directions. qRT-PCR results are represented as relative quantification normalized against internal control (GAPDH).

### Transfection of miR-9-5p mimics in BEAS-2B cells

BEAS-2B cells were transfected with 20 nM of miR-9-5p mimics (Integrated DNA Technologies, Coralville, Iowa, USA) using lipofectamine® RNAiMAX transfection reagent (ThermoFisher Scientific, Cat # 13778075) and Opti-MEM^TM^ reduced-serum medium (ThermoFisher Scientific, Cat # 31985062) according to manufacturer’s instruction. 24 hours post-transfection, cells were treated with TGF-β1 (10 ng/ml) and harvested 16 hours after treatment to quantify mRNA expression by qRT-PCR analysis.

### Statistical analysis

Unless otherwise mentioned, data were expressed as mean ± SEM from NHBE ALI cultures from at least three lungs. Statistical analysis was carried out and the data were subjected to statistical analysis using unpaired t-tests for two groups or ANOVA followed by Tukey Kramer honestly significant difference test for multiple comparisons as appropriate. Values of P < 0.05 were considered significant.

## Supplementary information

Supplementary Information
