# Supplementary material for: TGF-β1 increases viral burden and promotes HIV-1 latency in primary differentiated human bronchial epithelial cells
Source: Sci Rep. 2019 Aug 29;9:12552. doi: 10.1038/s41598-019-49056-6 (PMC6715689; doi:10.1038/s41598-019-49056-6)
Supplement: Supplementary file 1 — Supplementary Information [file 41598_2019_49056_MOESM1_ESM.pdf]

TGF-β1 increases viral burden and promotes HIV-1 latency in primary differentiated human bronchial epithelial cells

Chinnapaiyan S[1]., Dutta RK[1]., Nair M[1]., Chand HS[1]., Rahman I[2]., and Unwalla HJ[1*].

*To whom correspondence should be addressed.

Hoshang Unwalla, PhD, Department of Immunology and Nano-Medicine, Herbert Wertheim College of Medicine, Florida International University, 11200 SW 8th street, AHC-1 # 421, Miami, FL 33199, USA. Tel.: 305-348-3442; Fax: 305-243-6992;

email: hunwalla@fiu.edu

1. Department of Immunology and Nano-Medicine, Herbert Wertheim College of Medicine, Florida International University, Miami, FL 33199, USA.

2. University of Rochester Medical Center, School of Medicine and Dentistry, Rochester, NY 14642, USA

SUPPLEMENTARY FIGURE 1

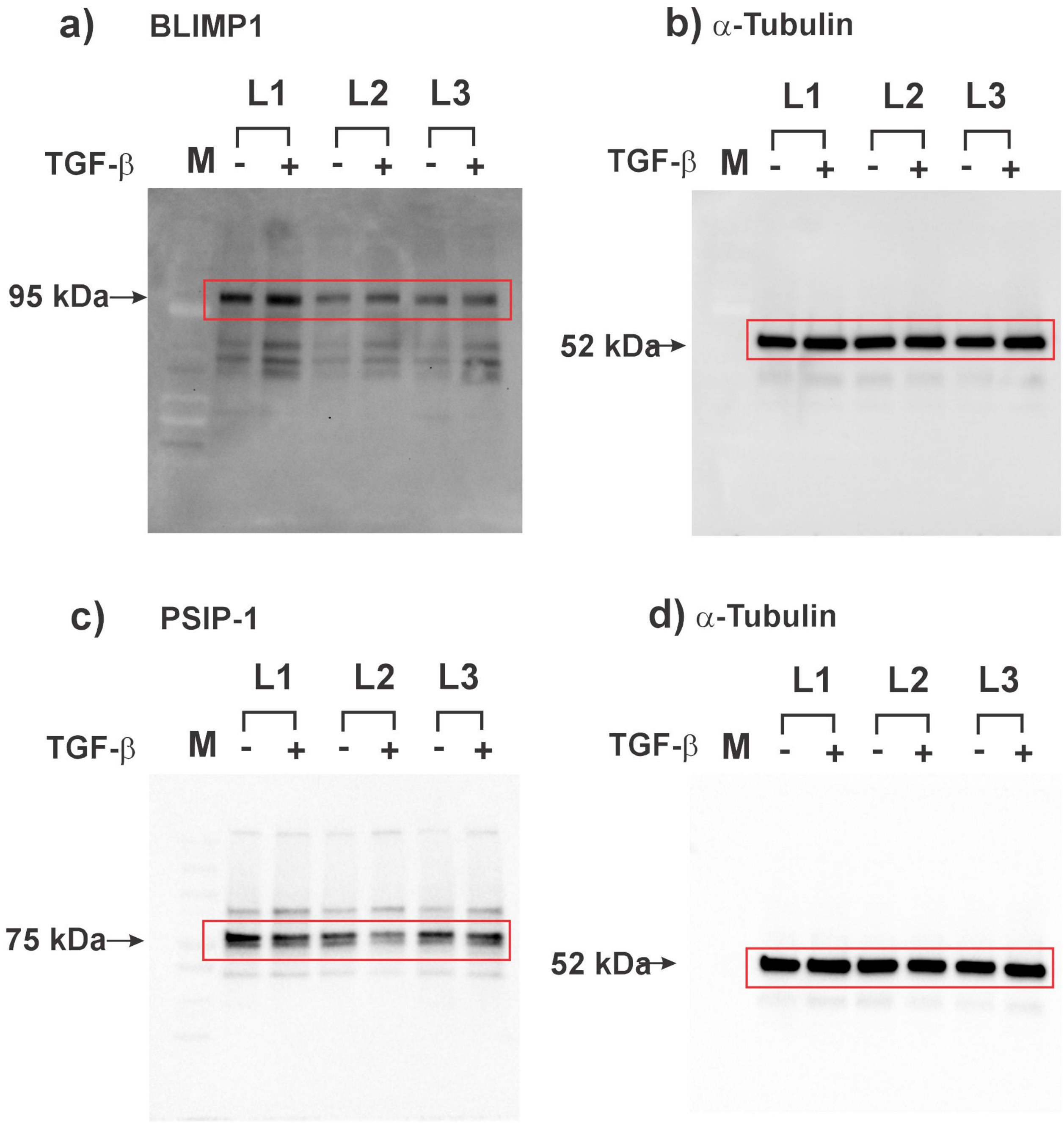

Full length western blot images. Panel a) BLIMP1; Panel b) PSIP-1. M: Protein marker;

Lane 2: Vehicle; Lane 3: TGF-β1; Lane 4: Vehicle; Lane 5: TGF-β1; Lane 6: Vehicle; Lane 7: TGF-β1. Red color highlighted bands were used for our manuscript. L1: Lung 1; L2: Lung 2; L3: Lung 3.

SUPPLEMENTARY FIGURE 2

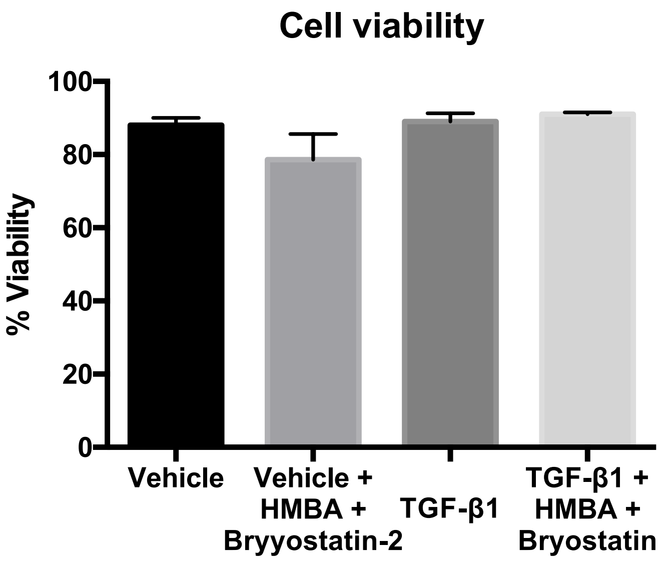

NHBE ALI cultures were infected with HIV BaL strain as described in Figure 1a. On day 8, Cell viability was determined using trypan blue staining. Only a marginal decrease in cell viability was observed using our infection and Treatment regimen.
